# Identification of Differentially Expressed lncRNAs and mRNAs in Children with Acquired Aplastic Anemia by RNA Sequencing

**DOI:** 10.1155/2020/8962090

**Published:** 2020-06-28

**Authors:** Shuanglong Lu, Xiaoxiao Song, Jing Chen, Xiaohong Qiao

**Affiliations:** ^1^Department of Pediatrics, Tongji Hospital, Tongji University School of Medicine, 389 Xincun Road, Shanghai 200065, China; ^2^Department of Hematology/Oncology, Shanghai Children's Medical Center, Shanghai Jiao Tong University School of Medicine, Shanghai 200127, China

## Abstract

**Background:**

The effects of long noncoding RNAs (lncRNAs) and their related messenger RNAs (mRNAs) remain unknown in children with acquired aplastic anemia (AA). The aim of this study is to screen key lncRNAs and mRNAs and investigate their potential roles in the pathology of acquired AA in children.

**Methods:**

RNA sequencing was performed to identify differentially expressed lncRNAs (DElncRNAs) and mRNAs (DEmRNAs) between blood samples of acquired AA children and healthy controls. *cis*-regulation, *trans*-regulation, competing endogenous (Ce) regulation networks of DElncRNAs and DEmRNAs were constructed. A literature search was performed to identify immune- or hematopoietic-related DElncRNA-DEmRNA pairs, and qPCR was conducted to validate the expression of the immune- or hematopoietic-related DElncRNA and DEmRNA.

**Results:**

60 DElncRNAs and 364 DEmRNAs were identified. 13 DElncRNAs were predicted to have 15 *cis*-regulated target DEmRNAs, 16 DElncRNAs might have 28 *trans*-regulated DEmRNAs, and 2 DElncRNAs might have 9 Ce-regulated DEmRNAs. After literature screen and qPCR validation, 6 immune- or hematopoietic-related DElncRNA-DEmRNA pairs in the networks above were identified as key RNAs in the pathology of acquired AA.

**Conclusion:**

This study revealed key lncRNAs in children with acquired AA and proposed their potential functions by predicting their target mRNAs, which lay the foundation for future study of potential effects of lncRNAs in children with acquired AA.

## 1. Introduction

Acquired aplastic anemia (acquired AA) is a life-threatening disorder in children characterized by pancytopenia and bone marrow failure. Successful use of immunosuppressive agents and hematopoietic stem cell transplantation (HSCT) in the treatment of acquired AA lead the way to understanding the pathology of acquired AA [1]. It is now widely acknowledged that at the cell level, the deficiencies of hematopoietic stem and progenitor cells (HSPCs), immune cell dysfunction, and abnormal bone marrow microenvironment are the main factors in the pathology of acquired AA [2, 3]. Besides, with the rapid developments in basic immunology and molecular biology techniques, a large number of studies have been carried out to explore the definitive mechanism at the molecular level in acquired AA. The messenger RNA (mRNA) expression profiles for CD34+ stem/progenitor cells [4, 5], T cells [6, 7], and mesenchymal stem/stromal (MSC) cells [8–10] in acquired AA have been described, and some mRNAs were identified to be involved in the pathology of acquired AA. Furthermore, microRNA (miRNA) expression profiles in acquired AA were also explored [11–13], and some miRNAs were identified to take part in the pathology of acquired AA. However, the long noncoding RNA (lncRNA) expression profiles and their role in children with acquired AA have not been described yet.

In the human genome, about 5%-10% sequences are transcribed, among which 10-20% are protein-coding RNAs and 80%-90% are non-protein-coding RNAs. lncRNAs are a kind of noncoding RNAs longer than 200 bp, and they can serve as signals, decoys, guides, and scaffolds in a large number of bioregulatory processes. Their biological role can be interpreted indirectly through the mRNAs which are regulated by *cis*-regulation, *trans*-regulation, or competing endogenous (Ce) regulation: the *cis*-regulation means that lncRNAs can affect the expression of their neighboring genes located at the same chromosome, the *trans*-regulation means that lncRNAs can also act on their target genes through a long-range manner such as conjunction with other transcription factors (TFs), and Ce regulation means that lnRNAs can act as sponges to compete for the miRNAs, hence reducing the miRNA's ability to interfere with the expression of target genes [14, 15].

It has been reported that lncRNAs are regulators of many immune processes and they participate in many immune-mediated disorders such as multiple sclerosis (MS) and systemic lupus erythematosus (SLE) [16, 17]. What is more, lncRNAs were also reported to regulate the hematopoietic stem cell development and play important roles in hematological disease [18]. As acquired AA is an immune-mediated hematological disease, it can be predicted that lncRNAs also play important roles in the pathology of acquired AA.

In this study, differentially expressed lncRNAs (DElncRNAs) and mRNAs (DEmRNAs) between acquired AA children and healthy controls were identified by RNA sequencing. The *cis*-, *trans*-, and Ce regulation networks were constructed to predict the DEmRNAs that might be regulated by DElncRNAs. Moreover, literature screen and quantitative real-time PCR (qPCR) validation were performed to identify immune- or hematopoietic-related DElncRNA-DEmRNA pairs, which may lay the foundation for future study of potential effects of lncRNAs in children with acquired AA.

## 2. Materials and Methods

### 2.1. Patients and Samples

Peripheral blood (PB) samples of 5 acquired AA children and 5 healthy controls were obtained at the Department of Pediatrics, Shanghai Tongji Hospital. After exclusion of any other marrow failure syndromes, the diagnosis of acquired AA was established by peripheral blood counts and bone marrow biopsy according to Camitta's criteria in the guideline [19]. Informed consent was obtained according to protocols approved by the Institutional Review Board of Shanghai Tongji Hospital affiliated to Tongji University. Student's *t*-test and Fisher's exact test were used to compare the basic characters of AA children and healthy controls.

### 2.2. RNA Extraction and Sequencing

Mononucleated cells of PB were separated by Solarbio R1010, and RNA was isolated according to the manufacturer's instructions. RNA sample sequencing of 5 acquired AA children and 5 healthy controls was performed separately based on the Illumina HiSeq 2000/2500 platform (Illumina, Inc., San Diego, CA, USA) with a 150 bp read length. The FASTQ sequence data were acquired from the RNA sequencing data. Reads with low quality were removed to obtain the clean reads.

### 2.3. Identification of DElncRNAs and DEmRNAs

Sequencing reads were aligned to the human genome (hg38) reference sequence, HTSeq was used, and the expression of mRNAs and lncRNAs was normalized. Reads Per Kilobase per Million (RPKM) of lncRNAs and mRNAs were calculated. DESeq2 was used for the differential expression analysis, and DEmRNAs and DElncRNAs were obtained with ∣log_2_FC | >1 and adj. *P* value < 0.05. By using the R package “pheatmap,” hierarchical clustering analysis of DEmRNAs and DElncRNAs was conducted.

### 2.4. Functional Annotation of DEmRNAs

To understand the biological functions and potential pathways of DEmRNAs, Gene Ontology (GO) functional annotation and Kyoto Encyclopedia of Genes and Genomes (KEGG) pathway enrichment analysis were performed and visualized by DAVID [20] and the R packages “clusterProfiler,” “enrichplot,” and “GOplot,” and adj. *P* value < 0.05 was considered to be significant.

### 2.5. *cis*-Regulated lnc-mRNA Network

To further explore the potential effects of DElncRNAs in children with acquired AA, the DElncRNA-DEmRNA coexpression networks were constructed. DElncRNA-DEmRNA pairs with an absolute value of the Pearson correlation > 0.9 and *P* < 0.01 were defined as coexpressed DElncRNA-DEmRNA pairs. The *cis*-regulated DEmRNAs were defined as follows: (1) DEmRNA loci were within a 100 kb window down- or upstream of the given DElncRNA and (2) DElncRNAs and DEmRNAs are coexpressed DElncRNA-DEmRNA pairs. The *cis*-regulated lnc-mRNA network was visualized by Cytoscape.

### 2.6. *trans*-Regulated lnc-TF-mRNA Network

For a *trans*-regulated network, we focused on the manner that lncRNAs play their functions via TFs. The DElncRNAs' coexpressed DEmRNAs were overlapping with TF target DEmRNAs in DAVID, using hypergeometric distribution to calculate the significance of this overlap, and adj. *P* value < 0.05 was considered to be significant. If the DElncRNAs' coexpressed DEmRNAs were overlapping with the target mRNAs of a given TF significantly, it meant that this TF might work with these DElncRNAs and these DEmRNAs could be the *trans*-regulated target of these DElncRNAs. The *trans*-regulated lnc-TF-mRNA network was constructed and visualized by Cytoscape.

### 2.7. Ce-Regulated lnc-micromRNA Network

Some lncRNAs might act as competing endogenous RNAs and influence the posttranscriptional regulation by regulating miRNA. miRNA-binding sites on DEmRNAs and DElncRNAs were predicted by software, and a Ce-regulated lnc-micromRNA network was constructed and visualized by Cytoscape.

### 2.8. Quantitative Real-Time PCR Validation

Blood samples of 5 acquired AA children and 5 healthy controls were used for qPCR validation, respectively. M-MLV reverse transcriptase was used for cDNA synthesizing. Subsequently, qPCR using SYBR Green assays was conducted in a total reaction volume of 10 *μ*l, including 0.5 *μ*l (10 *μ*M) PCR forward primer and 0.5 *μ*l (10 *μ*M) PCR reverse primer, 2 *μ*l CDNA, 5 *μ*l 2 × Master Mix, and 2 *μ*l double-distilled water. The qPCR reaction conditions were denaturation at 95°C for 10 min, followed by 40 cycles of 95°C (10 s) and 60°C (60 s). GAPDH was used as a reference. The relative expression level of each RNA was calculated using the 2^-*ΔΔ*Ct^ method, Student's *t*-test was applied to compare the expression levels of two groups, and *P* < 0.05 was considered to be significant. The primers are shown in Supplement Table 1.

## 3. Results

### 3.1. Clinical Characteristics of 5 Acquired AA Patients and 5 Healthy Controls

The clinical characteristics of 5 acquired AA patients and 5 healthy controls are listed in Table 1. No significant differences were found in age and gender between the two groups.

### 3.2. DEmRNAs and DElncRNAs between Acquired AA Children and Healthy Controls

**Figure 1:**
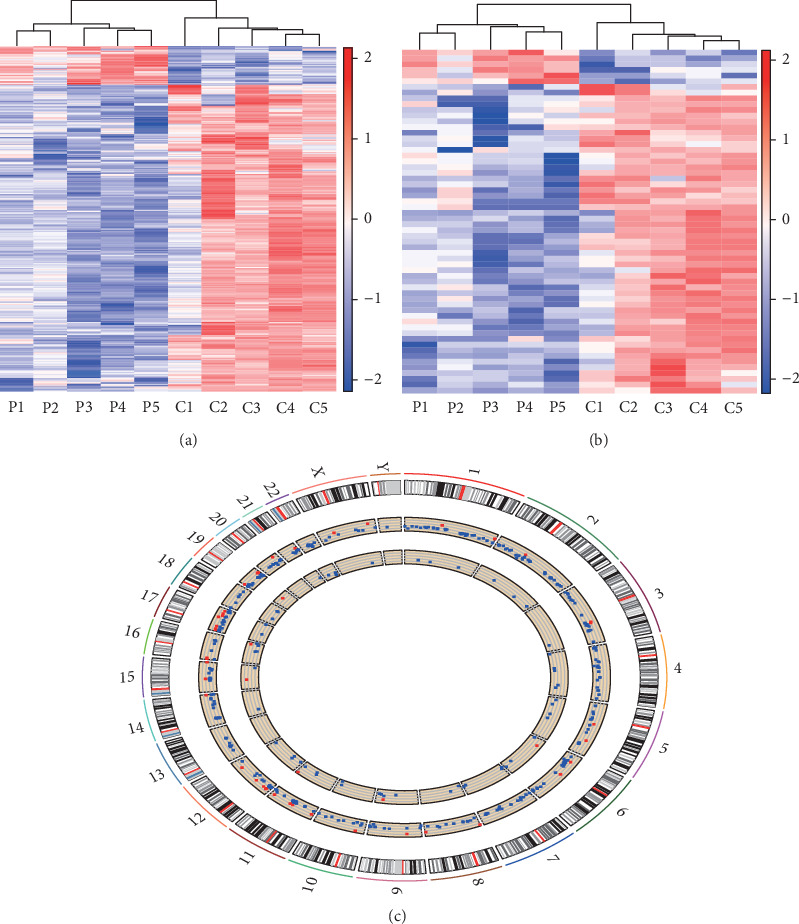
DEmRNAs and DElncRNAs between acquired AA children and healthy controls. (a and b) Heatmaps of DEmRNAs and DElncRNAs. P represents acquired AA children, and C represents healthy controls. The colors in the heatmap represent normalized gene expression values, with high expression values being colored in red and low expression values being colored in blue. (c) Distribution of DEmRNAs and DElncRNAs on chromosomes. The outer layer cycle is the chromosome map of the human genome hg38. The larger inner layer and smaller inner layer represent the distribution of DEmRNAs and DElncRNAs on different chromosomes, respectively. The red and blue colors represent the up- and downregulated RNAs, respectively.

A total of 364 DEmRNAs (41 upregulated and 323 downregulated DEmRNAs) and 60 DElncRNAs (6 upregulated and 54 downregulated DElncRNAs) were identified. The heatmap and hierarchical clustering analysis of DEmRNAs and DElncRNAs are depicted in Figures 1(a) and 1(b), respectively. Moreover, the distribution of DEmRNAs and DElncRNAs on chromosomes is shown in Figure 1(c).

### 3.3. Functional Annotation of 364 DEmRNAs

**Figure 2:**
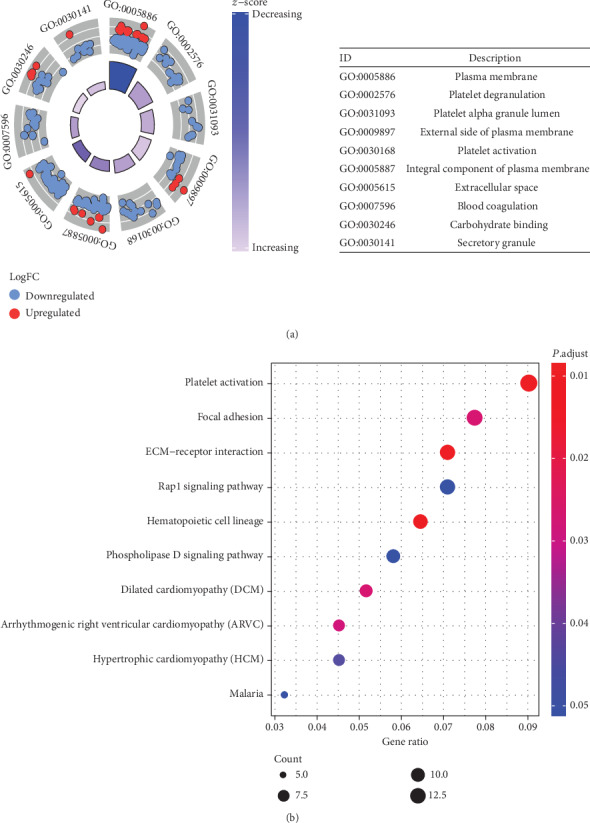
Functional annotation of 364 DEmRNAs. (a) Top 10 GO terms of 364 DEmRNAs and the DEmRNAs enriched in each term. The red and blue colors represent the up- and downregulated RNAs, respectively. (b) Top 10 KEGG terms of 364 DEmRNAs.

Platelet-related terms, platelet activation, platelet degranulation, and platelet alpha granule lumen, were enriched GO terms in acquired AA children (Figure 2(a)). Platelet activation and hematopoietic cell lineage were enriched KEGG pathways in acquired AA children (Figure 2(b)).

### 3.4. *cis*-Regulated lnc-mRNA Network

**Figure 3:**
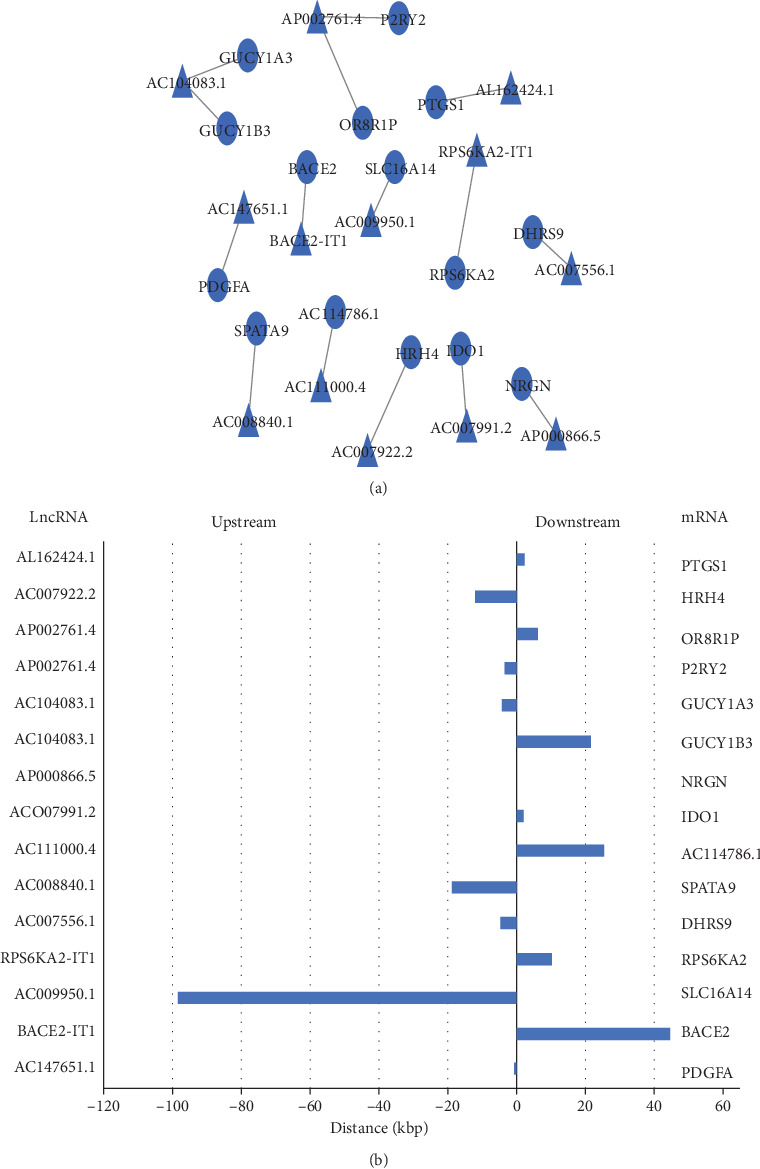
*cis*-regulation lnc-mRNA network. (a) DElncRNAs and their potential *cis*-regulated nearby DEmRNAs are shown in the network. The blue triangles represent the downregulated lncRNAs. The blue circles represent the downregulated DEmRNAs. (b) The distances between DElncRNAs and their *cis*-regulated DEmRNAs are presented. The left vertical axis shows the DElncRNAs, and the right vertical axis displays DEmRNAs.

A total of 15 *cis*-regulated DElncRNA-DEmRNA pairs were identified, including 13 DElncRNAs and 15 DEmRNAs. All paired DElncRNAs and DEmRNAs were downregulated RNAs in acquired AA children. The *cis*-regulated lnc-mRNA network is shown in Figure 3(a), and the distance between DElncRNAs and DEmRNAs in the network is shown in Figure 3(b).

### 3.5. *trans*-Regulated lnc-TF-mRNA Network and Ce-Regulated lnc-micromRNA Network

**Figure 4:**
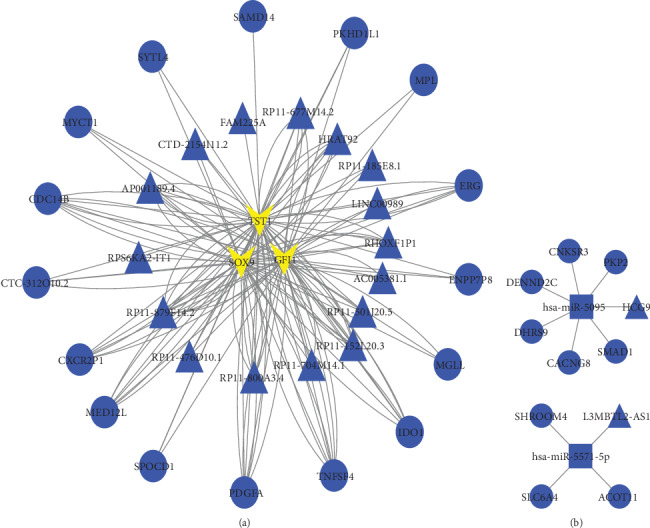
Trans-regulated lnc-TF-mRNA network and Ce-regulated lnc-micromRNA network. (a) DElncRNAs and their potential *trans*-regulated DEmRNAs and transcriptional factors involved in the *trans*-regulation are shown in the network. The blue triangles represent the downregulated lncRNAs. The blue circles represent the downregulated DEmRNAs. The yellow arrows represent TF. (b) DElncRNAs and their potential Ce-regulated DEmRNAs and the miRNAs targeting both RNAs are shown in the network. The blue triangles represent the downregulated lncRNAs. The blue circles represent the downregulated DEmRNAs. The blue squares represent miRNAs.

Twenty-eight DEmRNAs may be *trans*-regulated targets of 16 DElncRNAs. Transcriptional factors SOX9, GFI1, and TST1 might be involved in the *trans*-regulation, and the lnc-TF-mRNA network is shown in Figure 4(a). Two DElncRNAs may indirectly regulate 9 DEmRNAs by competing for hsa-miR-5095 and hsa-miR-5571-5p. The lnc-micromRNA network is shown in Figure 4(b).

### 3.6. Identification of Key DElncRNA-DEmRNA Pairs and qPCR Validation

**Figure 5:**
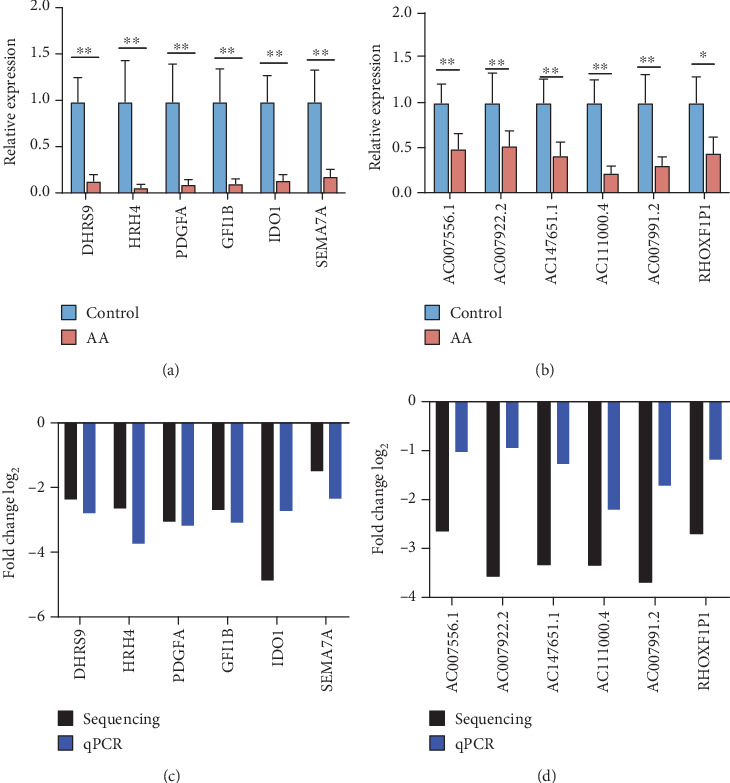
Validation for the expression of 6 immune- or hematopoietic-related DElncRNA-DEmRNA pairs by quantitative RT-PCR. The relative expression levels of 6 DEmRNAs (a) and 6 DElncRNAs (b) comparing AA patients and healthy controls. Data are presented as mean ± SD (^∗∗^*P* < 0.01, ^∗^*P* < 0.05). (c, d) Comparison between RNA sequencing and qPCR reveals a good correlation of two methods. The heights of the columns represent the log_2_-transformed fold changes computed from RNA sequencing and qPCR.

After literature screen of the above *cis*-, *trans*-, and Ce regulation networks, 6 immune or hematopoietic disease-related DEmRNAs and their paired DElncRNAs are listed in Table 2. To confirm the reliability of our sequencing data, the expression level of 6 immune or hematopoietic disease-related DElncRNA-DEmRNA pairs was validated by qPCR (Figure 5). The qPCR results were consistent with the sequencing data and showed the same trends of down regulation for each RNA.

## 4. Discussion

lncRNAs are >200 bp non-protein-coding transcripts that function as RNA molecules. Genome-wide transcriptome studies have led to the discovery of thousands of noncoding RNAs. It has been demonstrated that lncRNAs are involved in the pathology of many diseases, including immune-mediated disorders [16, 17] and hematological diseases [18]. A previous study has demonstrated that lncRNA TDRG1 may be involved in the proliferation of bone marrow mesenchymal stem cells in AA patients [21]. However, the expression profiles of lncRNAs and the potential targets or functions of lncRNAs in children with acquired AA remain unknown. Hence, in this study, we systematically screened the expression profiles of lncRNAs and mRNAs in acquired AA children and healthy controls.

Functional annotation of DEmRNAs revealed that dysregulated genes of acquired AA children are enriched in platelet-related terms including platelet activation, blood coagulation, and hematopoietic cell lineage. As acquired AA is usually manifested as pancytopenia, especially thrombocytopenia, these platelet function-related DEmRNAs and coagulation-related DEmRNAs may work as negative feedbacks and compensate the thrombocytopenia in some degree.

Unlike miRNAs, solely basing on lncRNAs' sequences to predict their function is difficult. Based on a previous study by Guttman et al. [22], we constructed a coexpression network of DElncRNAs and DEmRNAs. According to this network, the *cis*-regulation, *trans*-regulation, and Ce regulation networks were constructed to comprehend the biological functions of DElncRNAs. After literature screen and qPCR validation, 6 immune- or hematopoietic-related DElncRNA-DEmRNA pairs in the networks were identified as key lncRNAs and mRNAs in the pathology of acquired AA.

For the immune-related genes, DHRS9 [23] was reported to be a specific marker of the human regulatory macrophage and HRH4 [24] can downregulate Th1-related chemokines. As acquired AA is an immune-mediated disease, these two DEmRNAs in our networks may be involved in the pathology of acquired AA. Our work showed that these two downregulated DEmRNAs can be regulated by lncRNA AC007556.1 and AC007922.2 in *cis*- and Ce regulation manners. Hence, we can conclude that lncRNA AC007556.1 and AC007922.2 may be involved in the pathology of acquired AA by regulating DHRS9 and HRH4.

For the hematopoietic-related genes, PDGFA [25] and GFI1B [26, 27] were crucial for the hematopoiesis and they may be related to acquired AA. Our work showed that these downregulated DEmRNAs can be regulated by lncRNAs AC147651.1 and AC111000.4 in *cis*- and *trans*-regulation manners. Hence, we can conclude that lncRNAs AC147651.1 and AC111000.4 may be involved in the pathology of acquired AA by regulating PDGFA and GFI1B.

What is more, IDO1 [28] and SEMA7A [29] were reported to be important in the immunomodulatory effect of mesenchymal stromal cells in acquired AA. In our study, IDO1 and SEMA7A were downregulated and they were shown to be *cis*- and *trans*-regulated by lncRNAs AC007991.2 and RHOXF1P1. We can also conclude that lncRNAs AC007991.2 and RHOXF1P1 may be involved in the pathology of acquired AA by regulating IDO1 and SEMA7A.

There are limitations in our study. Firstly, our study is only a small sample size study which needs further validation. Another limitation is that we merely predict the potential link between lncRNAs and their target mRNAs and the definite connections between them could not be confirmed by the present study. Further study will be continued to validate the *cis*-, *trans*-, and Ce regulation networks.

## 5. Conclusion

In summary, our study describes the expression profiles of lncRNAs and mRNAs in acquired AA children by RNA sequencing. The *cis*-, *trans*-, and Ce regulation networks of DElncRNAs and DEmRNAs were identified, and 6 immune- or hematopoietic-related DElncRNA-DEmRNA pairs were identified as key RNAs in the pathology of acquired AA, which lay the foundation for future exploration of potential effects of lncRNAs and their target mRNAs in children with acquired AA.

## Figures and Tables

**Table 1:** Clinical characteristics of 5 acquired AA patients and 5 healthy controls.

Group	ID	Age (year)^∗^	Gender^∗^	Diagnosis	HB (g/l)	WBC (10^9^/l)	Neutrophils (10^9^/l)	Platelet (10^9^/l)
Patients	1	6	M	NSAA	110	3	0.9	25
	2	10	M	NSAA	70	3.5	0.9	40
	3	8	F	SAA	56	3	0.4	5
	4	2.1	F	SAA	75	2.3	0.4	11
	5	3.8	F	SAA	71	3.35	0.1	2

Healthy controls	1	8	M	—	137	7.8	5.3	211
	2	4	F	—	142	6.4	3.4	202
	3	1.7	M	—	121	7.2	3.6	183
	4	6	M	—	142	8	5.5	316
	5	2.7	F	—	124	6	2.1	204

^∗^No statistical differences were found between patients and healthy controls (age and gender).

**Table 2:** Six immune- or hematopoietic-related DElncRNA-DEmRNA pairs.

mRNA	Log_2_FC	lncRNA	Regulation type	mRNA function
DHRS9	-2.37	AC007556.1	Cis/Ce	Specific and stable marker of the human regulatory macrophage
HRH4	-2.64	AC007922.2	Cis	Downregulation of Th1-related chemokines
PDGFA	-3.05	AC147651.1	Cis	Promotes erythropoiesis and the proliferation of multipotent hematopoietic progenitors
GFI1B	-2.69	AC111000.4	Trans	Essential protein for the normal development of the megakaryocyte lineage
IDO1	-4.87	AC007991.2	Cis	Triggers an immunosuppressive state with increased FoxP3+ Treg cells
SEMA7A	-1.49	RHOXF1P1	Trans	Enhances the immunomodulatory properties of MSCs

## Data Availability

The clinical data of our patients are shown in Table 1.

## Supplementary Materials

Supplementary Materials Supplement Table 1: the clinical features of the patients in our manuscript.

## References

[1] Young N. S. (2018). Aplastic anemia. *The New England Journal of Medicine*.

[2] Wang L., Liu H. (2019). Pathogenesis of aplastic anemia. *Hematology*.

[3] Liu C., Sun Y., Shao Z. (2019). Current concepts of the pathogenesis of aplastic anemia. *Current Pharmaceutical Design*.

[4] Zeng W., Chen G., Kajigaya S. (2004). Gene expression profiling in CD34 cells to identify differences between aplastic anemia patients and healthy volunteers. *Blood*.

[5] Fischer U., Ruckert C., Hubner B. (2012). CD34+ gene expression profiling of individual children with very severe aplastic anemia indicates a pathogenic role of integrin receptors and the proapoptotic death ligand TRAIL. *Haematologica*.

[6] Zeng W., Kajigaya S., Chen G., Risitano A. M., Nunez O., Young N. S. (2004). Transcript profile of CD4^+^ and CD8^+^ T cells from the bone marrow of acquired aplastic anemia patients. *Experimental Hematology*.

[7] Franzke A., Geffers R., Hunger J. K. (2006). Identification of novel regulators in T-cell differentiation of aplastic anemia patients. *BMC Genomics*.

[8] Li J., Yang S., Lu S. (2012). Differential gene expression profile associated with the abnormality of bone marrow mesenchymal stem cells in aplastic anemia. *Plos One*.

[9] Chao Y. H., Wu K. H., Chiou S. H. (2015). Downregulated CXCL12 expression in mesenchymal stem cells associated with severe aplastic anemia in children. *Annals of Hematology*.

[10] Huo J., Zhang L., Ren X. (2020). Multifaceted characterization of the signatures and efficacy of mesenchymal stem/stromal cells in acquired aplastic anemia. *Stem Cell Research & Therapy*.

[11] Sun Y. X., Li H., Feng Q. (2017). Dysregulated miR34a/diacylglycerol kinase *ζ* interaction enhances T-cell activation in acquired aplastic anemia. *Oncotarget*.

[12] Hosokawa K., Muranski P., Feng X. (2015). Identification of novel microRNA signatures linked to acquired aplastic anemia. *Haematologica*.

[13] Shao Y. Q., Dong H. Y., Ge M. L. (2018). Differential expression profiles of microRNAs between de novo and complete response severe aplastic anemia. *Zhongguo Shi Yan Xue Ye Xue Za Zhi*.

[14] Mercer T. R., Dinger M. E., Mattick J. S. (2009). Long non-coding RNAs: insights into functions. *Nature Reviews Genetics*.

[15] Rinn J. L., Chang H. Y. (2012). Genome regulation by long noncoding RNAs. *Annual Review of Biochemistry*.

[16] Atianand M. K., Caffrey D. R., Fitzgerald K. A. (2017). Immunobiology of long noncoding RNAs. *Annual Review of Immunology*.

[17] Uthaya Kumar D. B., Williams A. (2020). Long non-coding RNAs in immune regulation and their potential as therapeutic targets. *International Immunopharmacology*.

[18] Dahariya S., Paddibhatla I., Kumar S., Raghuwanshi S., Pallepati A., Gutti R. K. (2019). Long non-coding RNA: classification, biogenesis and functions in blood cells. *Molecular Immunology*.

[19] Marsh J. C. W., Ball S. E., Cavenagh J. (2009). Guidelines for the diagnosis and management of aplastic anaemia. *British Journal of Haematology*.

[20] Jiao X., Sherman B. T., Huang D. W. (2012). DAVID-WS: a stateful web service to facilitate gene/protein list analysis. *Bioinformatics*.

[21] Jiang S., Xia M., Yang J. (2015). Novel insights into a treatment for aplastic anemia based on the advanced proliferation of bone marrow-derived mesenchymal stem cells induced by fibroblast growth factor 1. *Molecular Medicine Reports*.

[22] Guttman M., Amit I., Garber M. (2009). Chromatin signature reveals over a thousand highly conserved large non- coding RNAs in mammals. *Nature*.

[23] Riquelme P., Amodio G., Macedo C. (2017). DHRS9 is a stable marker of human regulatory macrophages. *Transplantation*.

[24] Mommert S., Ratz L., Stark H., Gutzmer R., Werfel T. (2018). The histamine H4 receptor modulates the differentiation process of human monocyte-derived M1 macrophages and the release of CCL4/MIP-1*β* from fully differentiated M1 macrophages. *Inflammation Research*.

[25] Chui C. M. Y., Li K., Yang M. (2003). Platelet-derived growth factor up-regulates the expression of transcription factors NF-E2, GATA-1 and c-Fos in megakaryocytic cell lines. *Cytokine*.

[26] Möröy T., Vassen L., Wilkes B., Khandanpour C. (2015). From cytopenia to leukemia: the role of Gfi1 and Gfi1b in blood formation. *Blood*.

[27] Kitamura K., Okuno Y., Yoshida K. (2016). Functional characterization of a novel GFI1B mutation causing congenital macrothrombocytopenia. *Journal of Thrombosis and Haemostasis*.

[28] Vidotto T., Saggioro F. P., Jamaspishvili T. (2019). PTEN‐deficient prostate cancer is associated with an immunosuppressive tumor microenvironment mediated by increased expression of IDO1 and infiltrating FoxP3+ T regulatory cells. *Prostate*.

[29] Fayyad-Kazan M., Najar M., Fayyad-Kazan H., Raicevic G., Lagneaux L. (2017). Identification and evaluation of new immunoregulatory genes in mesenchymal stromal cells of different origins: comparison of normal and inflammatory conditions. *Medical Science Monitor Basic Research*.

