# Supplementary material for: Identification of Differentially Expressed lncRNAs and mRNAs in Children with Acquired Aplastic Anemia by RNA Sequencing
Source: Biomed Res Int. 2020 Jun 28;2020:8962090. doi: 10.1155/2020/8962090 (PMC7338984; doi:10.1155/2020/8962090)
Supplement: Supplementary Materials — Supplement Table 1: the clinical features of the patients in our manuscript. [file 8962090.f1.docx]

**Supplement Table 1. Primers used for the qPCR validation of** **immune or hematopoietic related DEmRNA and DElncRNA .**

| ID | Primers |
| --- | --- |
| DHRS9-qPCR-F | CTTCTGGATGTGACCGACCC |
| DHRS9-qPCR-R | TCTAGTGTCAGCCAGTCAGT |
| HRH4-qPCR-F | AACTTGGCCATCTCTGACTTC |
| HRH4-qPCR-R | TAGTGAGCCAAAATACACAGAT |
| PDGFA-qPCR-F | CGGATACCTCGCCCATGTTCTG |
| PDGFA-qPCR-R | CTTCTCGGGCACATGCTTAGTGG |
| GFI1B-qPCR-F | AGAGAGGCTTTGCAGTTCCC |
| GFI1B-qPCR-R | GGTTCATCTTCCTGCACACGG |
| IDO1-qPCR-F | GCTTTGCTCTGCCAAATCCACA |
| IDO1-qPCR-R | TCCCAGAACTAGACGTGCAAGG |
| SEMA7A-qPCR-F | CACGGTTGCCTCATGTCCCGAGA |
| SEMA7A-qPCR-R | TTTGGGGTTGGGACACTCCT |
| AC007556.1-qPCR-F | TTTCTCCTAGGTATGGCAAC |
| AC007556.1-qPCR-R | CATCTCTGGTTCATAATTCAGGC |
| AC007922.2-qPCR-F | CAGCCCCCCGAGCAACTG |
| AC007922.2-qPCR-R | TCTTCCATGGGAGCTCCACA |
| AC147651.1-qPCR-F | TTTCATGGACACTCACGCATC |
| AC147651.1-qPCR-R | TTCTTGAAGCCTCCAAACATCC |
| AC111000.4-qPCR-F | TGCCTATGACAGGACTAACAGA |
| AC111000.4-qPCR-R | TTTTGTCAGTATTCACACGAAG |
| AC007991.2-qPCR-F | CATGGATACGGAGAGTGATC |
| AC007991.2-qPCR-R | ATTGTACCATTGATAAGTACATCCTT |
| RHOXF1P1-qPCR-F | CCAGAGGACCCCAGCGCAGA |
| RHOXF1P1-qPCR-R | TCATCGTCTCCTCCAGCTTCAGT |
